# Establishment and application of isothermal multiple-self-matching-initiated amplification (IMSA) in detecting Type II heat-labile enterotoxin of *Escherichia coli*

**DOI:** 10.1371/journal.pone.0216272

**Published:** 2019-05-02

**Authors:** Wenxin Liu, Chaowen Yuan, Liguo Zhang, Yufei Feng

**Affiliations:** 1 Affiliated Central People’s Hospital of Zhanjiang, Zhanjiang, Guangdong, China; 2 College of Life and Health Sciences, Northeastern University, Shenyang, Liaoning, China; 3 Center for Animal Disease Emergency of Liaoning province, Shenyang, Liaoning, China; 4 Affiliated Hospital of Guangdong Medical University, Zhanjiang, Guangdong, China; The Pennsylvania State University, UNITED STATES

## Abstract

Enterotoxigenic *Escherichia coli* (ETEC) constitutes a major cause of diarrhea in young children and animals, particularly in poor regions of the world, as well the traveler’s diarrhea in adult individuals. Type II heat-labile enterotoxin (LT-II) from ETEC can cause profuse watery diarrhea, posing a potential threat to public health and animal husbandry. In the present study, isothermal multiple-self-matching-initiated amplification (IMSA) was established to rapidly detect LT-II producing ETEC. The specificity and sensitivity were assessed, and clinical samples were tested. The established IMSA method had good specificity for the detection of LT-II gene with a limit of detection of 25 CFU/mL, i.e. 2 times higher than that of real-time PCR and other two isothermal amplifications (loop-mediated isothermal amplification, LAMP and cross-primer isothermal amplification, CPA). Meanwhile, in 103 clinical *Escherichia coli* strains isolated from diarrhea samples, 9 strains with LT-II^+^ gene were detected (8.73%), corroborating real-time PCR, LAMP and CPA data. Therefore, the IMSA technology applied for the detection of LT-II producing ETEC has a good application prospect for screening clinical samples in primary medical units or common laboratories.

## Introduction

Enterotoxigenic *Escherichia coli* (ETEC) is the most important etiologic agent of traveler’s diarrhea as well as diarrhea in poor regions of the world, especially in children [1]. For pathogenicity, ETEC first adheres to intestinal epithelial cells, using surface adhesins also referred to as fimbriae or pili, and produces distinct combinations of heat stable (ST) and heat-labile (LT) enterotoxins [2]. Two LT families (LT-I and LT-II) have been reported, that differ by antigenicity and receptor binding specificity. LT-II is associated with *E*. *coli* isolates from human, animal, food and water samples, and potentially cause disease in humans, calves and pigs [3].

Previously, ETEC detection was based on animal experiments and cell culture assays requiring specific antibodies. Subsequently, enzyme-linked immunosorbent assay (ELISA) and membrane-based DNA hybridization methods, which are faster and easy to carry out, gained popularity. In recent years, PCR based techniques have been developed to improve ETEC detection[4, 5]. Usage of these techniques are limited due to need for proper laboratory setting and experienced and knowledgeable personnel.

Isothermal amplification is superior to traditional PCR in terms of convenience, since reactions are performed at a fixed temperature, with no need for sophisticated equipment. Different isothermal -amplification techniques have been reported [6]. A decade ago loop-mediated isothermal amplification (LAMP) was proposed for fast, specific and efficient amplification of target genes at fixed temperatures [7]. Such methods have been used to detect many pathogens, e.g. viruses [8]. Cross-priming amplification (CPA) is a rapid detection technique [9], which is performed by DNA polymerase strand displacement activity, not requiring initial denaturation or a nicking enzyme [10]. With 5–6 primers, sensitivity and specificity can rise within 1h under isothermal conditions [11]. Isothermal multiple self-matching initiated amplification (IMSA) represents a novel isothermal amplification method resembling LAMP, using a strand displacement DNA polymerase for detection. IMSA uses three primer pairs: 1 forward and 1 reverse stem primers (SteF and SteR, respectively); 2 pairs of hybrid nested primers [12]. IMSA generates many self-matching structures (SMSs), i.e. single-stranded DNA products produced by the hybrid primers. In addition, IMSA can provide simple and fast DNA detection with lower limit of detection [13].

In the present study, an IMSA method for the LT-II gene of ETEC was developed and compared with LAMP, CPA and real-time PCR, which is a good tool for rapidly and sensitively detecting LT-II producing ETEC. The possibility of detecting LT-II positive ETEC isolated from both clinical and environmental sources should help identify and initiated by LT-II producing ETEC.

## Materials and methods

### Bacteria

Fourteen bacterial strains (6 *E*. *coli* and 8 non-*E*. *coli* strains; Table 1) were assessed in specificity assays. Strain OS-1 (LT-II^+^) was employed for standardization, technique optimization, sensitivity assessment and clinical specimen detection. From January 2016 and December 2017, a total of 218 clinical fecal specimens (136 from suckling pigs with diarrheal and 82 from cattle with diarrheal) were collected from different farms with diarrhea in the northeast of China, which were streaked on Mac-Conkey agar plates (Oxoid, Basingstoke, England) and incubated at 37°C overnight. The biochemically confirmed 103 isolated strains of *Escherichia coli* (indole production, citrate utilization, MR positive, VP negative and hydrogen sulfate production, etc)[14] were stored in LB (Luria-Bertani) (Mast group Ltd, Merseyside, UK) with 20% glycerol at—70°C until needed for DNA extraction.

**Table 1:** Bacterial strains.

Bacterial strain	Source
OS-1 (LT-II^+^)	The Affiliated Hospital of Guangdong Medical University
OS-6 (LT-II^+^)	The Affiliated Hospital of Guangdong Medical University
*Aeromonas hydrophila* ATCC 7966	American Type Culture Collection (ATCC)
*Clostridium perfringens* ATCC13124	ATCC
*Salmonella enteritidis* ATCC 13076	ATCC
*Salmonella typhimurium* ATCC 13311	ATCC
*Staphylococcus aureus* ATCC 25923	ATCC
*Staphylococcus aureus* ATCC 29213	ATCC
*Yersinia enterocolitica* ATCC 23715	ATCC
*Vibrio parahaemolyticus* ATCC 27519	ATCC
*Enterotoxigenic E*. *coli* C83920 (Sta^+^)	China Institute of Veterinary Drug Control (CIVDC)
*Enterotoxigenic E*. *coli* C44498 (Stx2e^+^)	CIVDC
*Enterotoxigenic E*. *coli* O157: H7 (Stx1+, Stx2^+^)	CIVDC
*Enterotoxigenic E*. *coli* C83903 (LT-I^+^, Stb^+^)	CIVDC

### Primers

**Fig 1:**
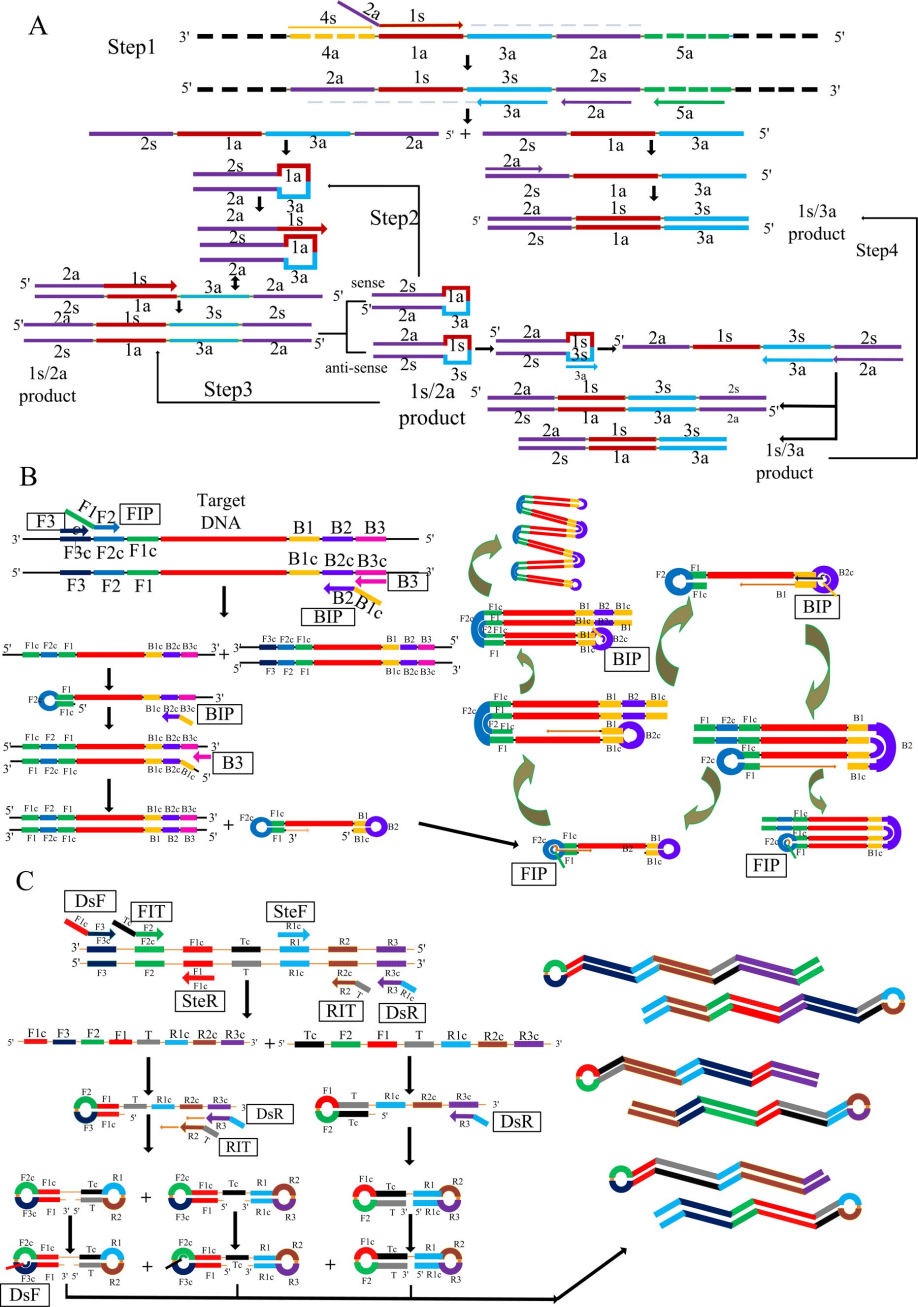
Schematic maps of CPA, LAMP and IMSA amplification reactions. A, Illustration of the CPA assay; B, Illustration of the LAMP assay; C, Illustration of the IMSA assay.

**Fig 2:**
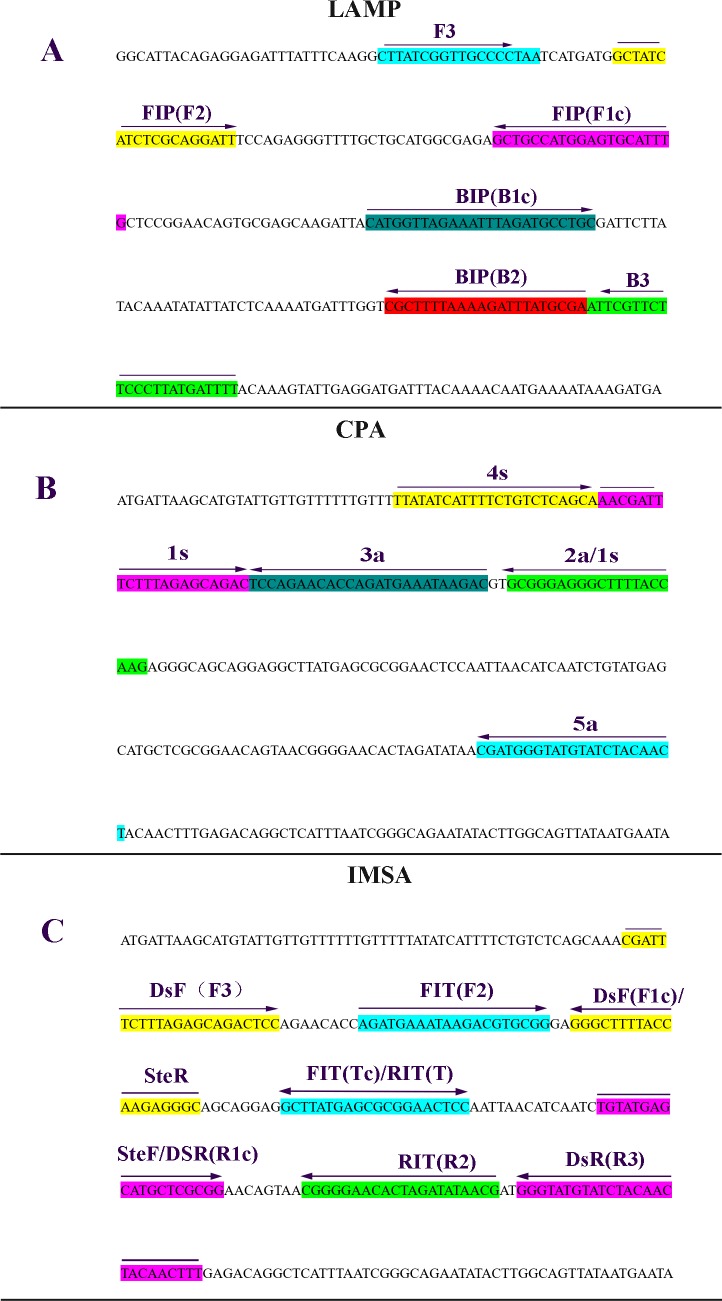
Primer locations on the LT-II gene. The direction of the arrows indicated the direction of primers extension and amplification. A, Primer design for LAMP; B, Primer design for CPA; C, Primer design for IMSA.

Oligonucleotide primers in CPA, LAMP and IMSA detection of ETEC were based on conserved LT-II gene sequences (GenBank accession number JQ031705.1). CPA primers were designed with two displacement primers and two cross primers using the Primer Premier 5.0 software. For LAMP, four (two each of outer [F3 and B3] and inner [FIP and BIP]) primers recognizing 4 different regions on each target sequence were obtained with Primer-Explorer V4 (http://primerexplorer.jp/e/). IMSA primers, comprising two each displacement and cross primers, were equally obtained with Primer Premier 5.0 (Figs 1 and 2). Table 2 summarizes all primers (Sangon Biotechnology Co., Ltd., Shanghai, China).

**Table 2:** Primers for LAMP, CPA, IMSA and Q-PCR assays.

	Primers Name	Primers Sequence (5'-3')
LAMP primers	F3	CTTATCGGTTGCCCCTAA
	B3	AAATCATAAGGGAAGAACGAAT
	FIP	CAAATGCACTCCATGGCAGC-GCTATCATCTCGCAGGATT
	BIP	CATGGTTAGAAATTTAGATGCCTGC-TCGCATAAATCTTTTAAAAGCG
CPA primers	1s	CTTGGTAAAAGCCCTCCCGC-AACGATTTCTTTAGAGCAGAC
	2a	CTTGGTAAAAGCCCTCCCGC
	3a	GTCTTATTTCATCTGGTGTTCTGGA
	4s	TTATATCATTTTCTGTCTCAGCA
	5a	AGTTGTAGATACATACCCATCG
IMSA primers	DSF	GCCCTCTTGGTAAAAGCCC-CGATTTCTTTAGAGCAGACTCC
	DsR	TGTATGAGCATGCTCGCGG-AAAGTTGTAGTTGTAGATACATACCC
	FIT	GGAGTTCCGCGCTCATAAGC-AGATGAAATAAGA
	RIT	CGTGCGGGCTTATGAGCGCGGAACTCC-CGTTATATCTAGTGTTCCCCG
	SteR	GCCCTCTTGGTAAAAGCCC
	SteF	TGTATGAGCATGCTCGCGG
QPCR Primers	Forward	TGTGAATGGTGTGTTAGGGCGGTAT
	Reverse	CTCCTCTGTAATGCCTGTTTCGCTG

### DNA extraction

*Escherichia coli* DNA templates were prepared from 3 mL of overnight cultures grown in LB (Luria-Bertani) at 37°C. *E*. *coli* were harvested, washed twice and resuspended in 100μL of TE (Tris +EDTA) buffer. Lysis was performed by boiling at 100°C for 10 min, and the supernatant was collected as the template.

### Reactions

**Fig 3:**
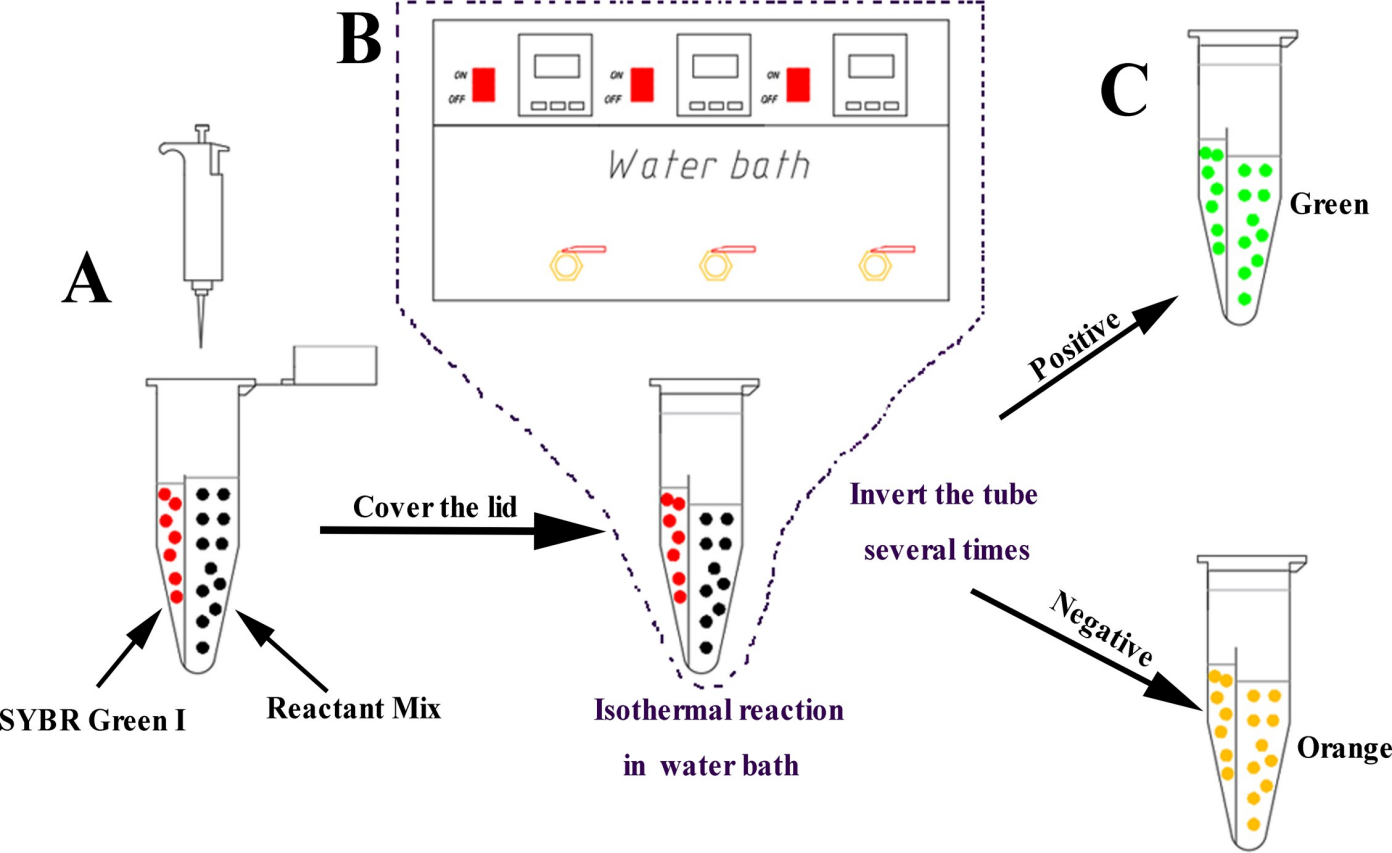
Schematic diagram of all isothermal reaction steps. A, SYBR Green I and the reaction mixture were added into a Hua-Feng tube (Guangzhou Hua-feng Biological Technology Co.,Ltd), respectively. B, Incubation at constant temperature for a period of time (45–90 min). C, Result determination.

LAMP, CPA and IMSA were performed in 25μL reactions containing 2μL 10 × *Bst* buffer, 8 U *Bst* DNA large-fragment polymerase (New England Biolabs, USA), 0.8μM dNTPs (TAKARA, China), 6μM MgCl_2_ (Sigma, USA), 0.5 M betaine (Sigma), 2μL target DNA and 1μL of SYBR Green I (2000×) (Solarbio, China) (Fig 3). For the LAMP assay, reaction primers included: FIP and BIP (40 pmoL) and F3 and B3 (10 pmoL). CPA reaction primers were: cross primer 1s (40 pmoL), primers 2a and 3a (20 pmoL) and displacement primers 4s and 5a (10 pmoL). The concentration of outer primers DsF/DsR was 10 pmoL; FIT/RIT and SteF/SteR had a concentration of 20 pmoL. The three amplifications were carried out at 60°C for 60 min followed by heating at 80°C (5 min) for reaction termination. The reactants were mixed with SYBR Green I and visualized by eye or under UV light. The resulting products were assessed by 2.0% agarose gel electrophoresis and staining with ethidium bromide (0.5 ug/ml).

### LAMP, CPA and IMSA assay optimization

The initial reaction conditions of the three isothermal methods were optimized for reaction temperature, Mg^2+^, *Bst* DNA polymerase and incubation time. A given optimization step started with previous optimization parameters; experiments were performed in triplicate. Temperature was optimized by assessing 60, 62, 64, 66, 68 and 70°C, respectively, as amplification temperature. Mg^2+^ concentrations assessed were set at 1.0, 2.0 and 3.0 mmol/L, while *Bst* DNA polymerase concentrations were 6, 8, 10 and 12 U, respectively; the incubation times tested were 30, 45, 60, 75, 90 and 120 min, respectively.

### Specificity of the assays

For specificity assays, DNA samples from the 14 bacterial strains (Table 1) were obtained as mentioned above. Then, 2μL of DNA template was submitted to IMSA, and results were compared with LAMP and CPA data. All experiments were repeated ≥ 3 times. Next, 5μL of the final product of each reaction was assessed by 2.0% agarose gel electrophoresis.

### Sensitivity analysis of the assays

For determining the limit of detection of IMSA, template DNA from OS-1 (LT-II^+^) (5×10^6^ CFU/mL) was prepared as described above and serially diluted ten-fold, and further adjusted to the dilutions of 25 CFU/mL, 15 CFU/mL and 5 CFU/mL. The reaction system was set as described above and reactions were performed at the adaptive temperature for 60 min, respectively. Then, 1μL of SYBR Green I was added to a Hua-Feng tube for direct visualization. Meanwhile, these samples were all tested by LAMP, CPA and real time PCR assays.

### Sample detection

To determine the validity and reliability of IMSA for detecting the LT-II gene of ETEC in clinical samples, a total of 103 *E*. *coli* isolates (63 fecal samples from piglets with diarrhea and 40 fecal specimens from cattle with diarrhea) were collected from 2016 to 2017. DNA was prepared from clinical samples and used as template, and result confirmation was by LAMP, CPA and real-time PCR.

## Results

### Amplification of the LT-II^+^ Gene by CPA, LAMP and IMSA

**Fig 4:**
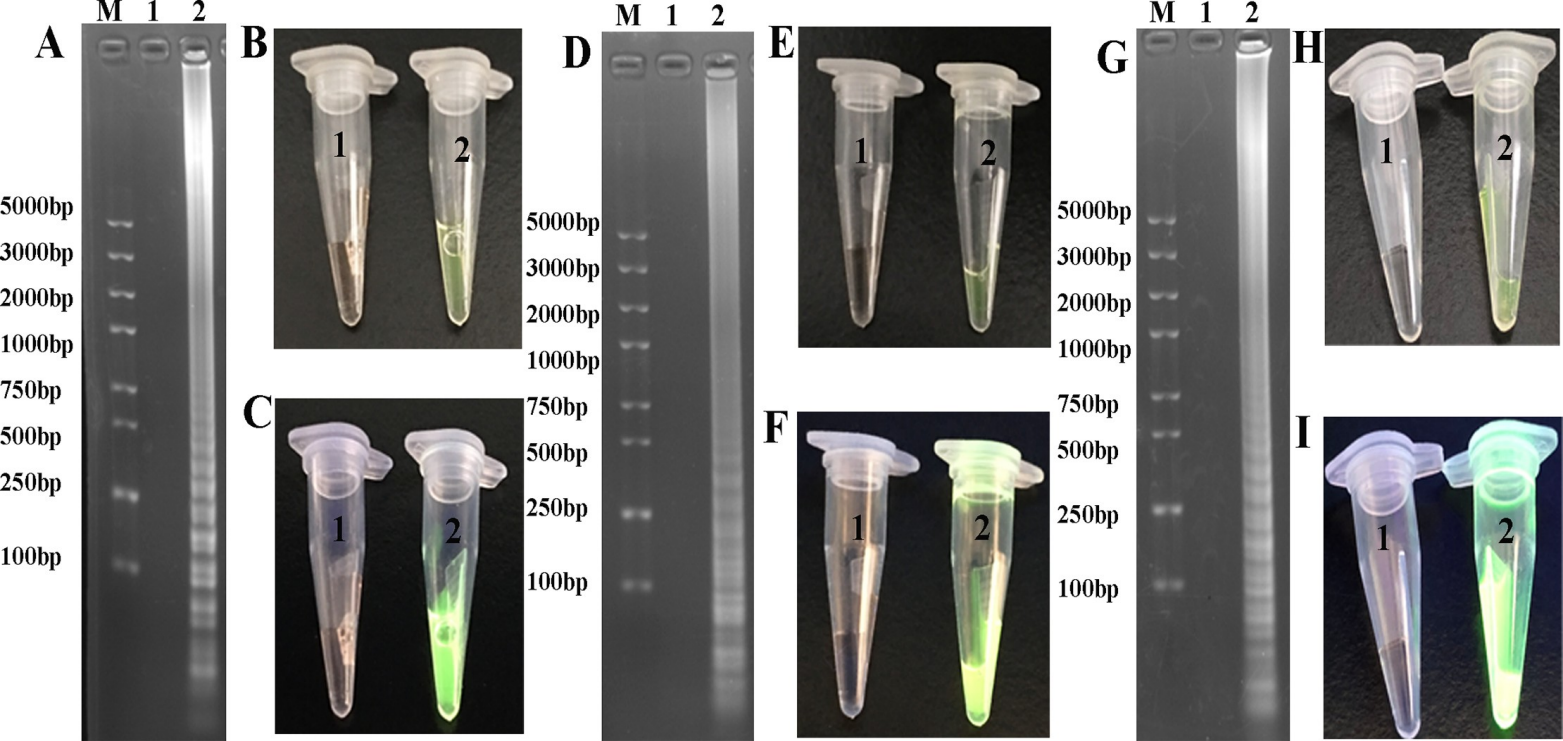
LAMP, CPA and IMSA amplification results. M, Trans 2K plus II DNA marker; 1, negative control; 2, Positive control. A, D and G, Agarose gel electrophoresis observation for LAMP, CPA and IMSA methods for LT-II gene. B, E and H, LAMP, CPA and IMSA reactions were observed upon addition of SYBR Green I, respectively. C, F and I, LAMP, CPA and IMSA reactions were visualized under UV light upon addition of SYBR Green I, respectively.

IMSA, LAMP and CPA yield mixed stem-loop DNAs of different sizes and cauliflower-like structures containing many loops resulting from annealing between alternately inverted repeats of the target sequence in the same strand. Upon reaction, the positive products yielded typical ladder-like bands on agarose gels, and reaction mixtures turned into green with SYBR Green I (Fig 4).

### Assay optimization

**Fig 5:**
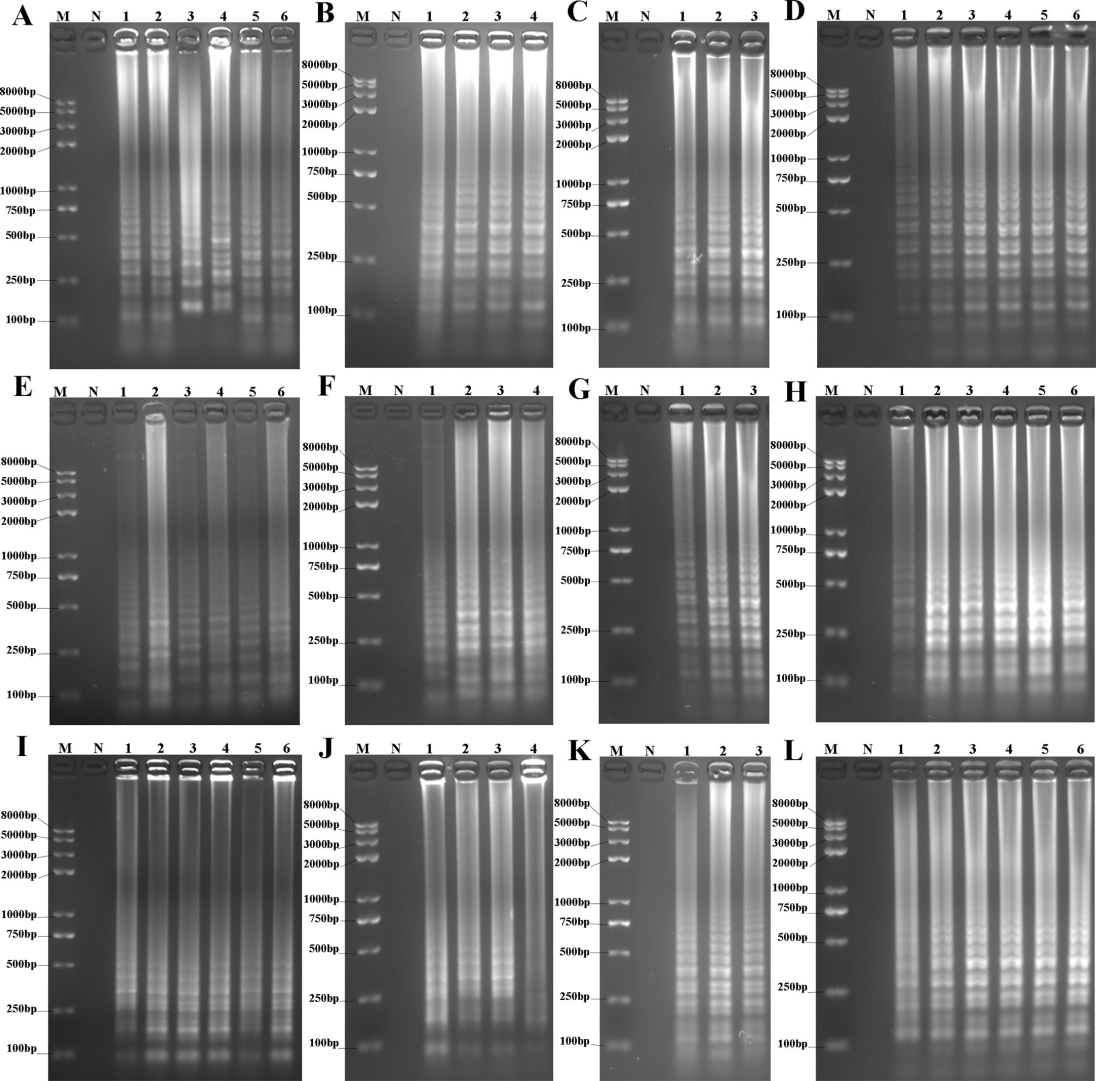
Optimization of various amplification conditions. A, E and I, Optimization of temperature for LAMP, CPA and IMSA, respectively. 1–6, Amplification products at 60, 62, 64, 66, 68 and 70°C, respectively. B, F and J, Optimization of *Bst* DNA enzyme concentration for LAMP, CPA and IMSA, respectively. 1–4, Amplification products for *Bst* DNA enzyme concentrations at 6, 8, 10 and 12 U, respectively; C, G and K, Optimization of Mg^2+^ concentration for LAMP, CPA and IMSA, respectively. 1–3, 1.0, 2.0 and 3.0 mM, respectively; D, H and L, Optimization of incubation time for LAMP, CPA and IMSA, respectively. 1–6, Amplification products at 30, 45, 60, 75, 90 and 120 min, respectively. M, Trans 2K plus II DNA marker; N, negative control.

Starting with initial CPA, LAMP and IMSA protocols, reaction temperatures, Mg^2+^ concentrations, *Bst* DNA large-fragment polymerase amounts and incubation times were optimized. As shown in Fig 5, the optimized LAMP conditions were: reaction temperature, 64°C (Fig 5A); *Bst* DNA large-fragment polymerase, 6 U (Fig 5B); Mg^2+^, 1.0 mM (Fig 5C); and reaction time, 60 min (Fig 5D). The optimized CPA conditions were 62°C for 90 min with 2.0 mM Mg^2+^ and 8 U *Bst* DNA large-fragment polymerase (Fig 5E–5H). The optimal IMSA reactions were incubated for 60 min at 60°C with 2.0 mM Mg^2+^ and 6 U *Bst* DNA large-fragment polymerase (Fig 5I–5L). The reactions were detected using 2% agarose gel electrophoresis.

### Specificities of various assays

**Fig 6:**
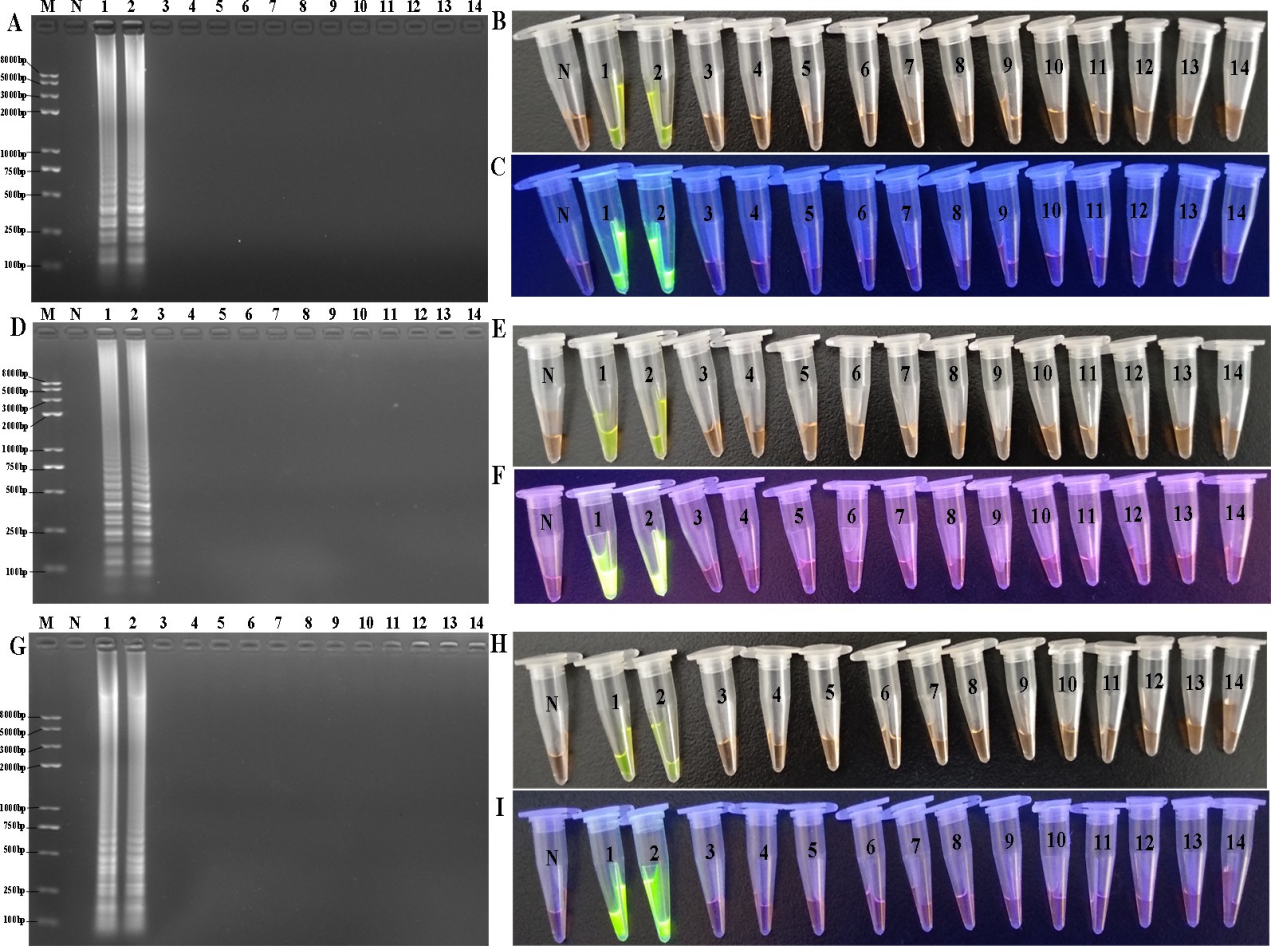
Specificities of LAMP, CPA and IMSA. A, D and G, Agarose gel electrophoresis analysis of LAMP, CPA and IMSA products; B, E and H, Direct visualization under natural light after staining with SYBR Green I for LAMP, CPA and IMSA reactions; C, F and I, Visualization under UV light after staining with SYBR Green I for LAMP, CPA and IMSA reactions; 1–2, OS-1 and OS-6, respectively. 4–14, C83920, C44498, O157:H7, C83903, ATCC7966, ATCC13076, ATCC13311, ATCC25923, ATCC29213, ATCC23715, ATCC27519 and ATCC13124, respectively. M, Trans 2K plus II DNA marker; N, negative control.

The detection specificity of IMSA was assessed with multiple *E*. *coli* and non-*E*. *coli* isolates (Table 1). Two LT-II^+^ strains were identified by IMSA, and no false positive DNA products were observed from the control strains, which was consistent with LAMP and CPA results (Fig 6).

### Sensitivities of various assays

**Fig 7:**
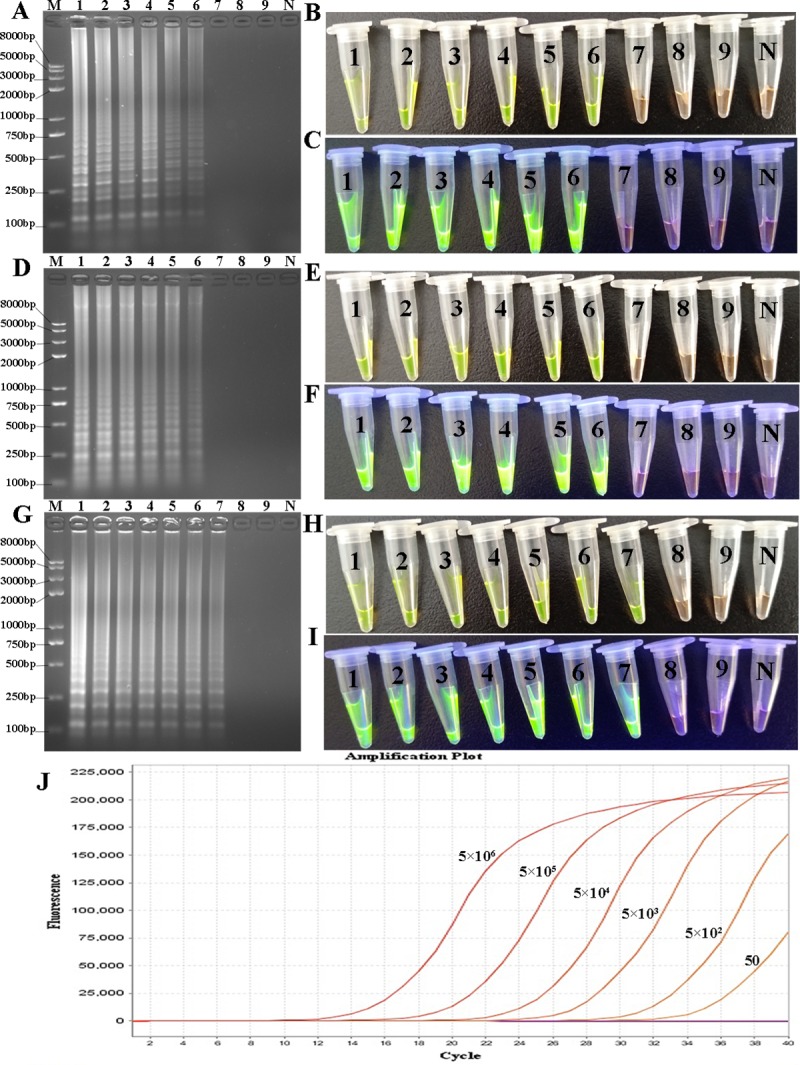
Sensitivities of LAMP, CPA, IMSA and real-time PCR. A, D and G, Agarose gel electrophoresis analysis of LAMP, CPA and IMSA reactions; B, E and H, Direct visualization under natural light after staining with SYBR Green I for LAMP, CPA and IMSA reactions; C, F and I, Visualization under UV light after staining with SYBR Green I for LAMP, CPA and IMSA reactions; J, real-time PCR reaction; 1–9, 5×10^6^, 5×10^5^, 5×10^4^, 5×10^3^, 5×10^2^, 5×10^1^, 25, 15 and 5 CFU/mL, respectively; M, Trans 2K plus II DNA marker; N, negative control.

Serial dilutions of total genomic DNA from OS-1 (LT-II^+^) were used to assess the sensitivities of LAMP, CPA and IMSA assays, respectively. The results indicated a limit of detection for IMSA of 25 CFU/tube, which was two times reduced as compared with that of the other two isothermal amplifications (LAMP and CPA) and real-time PCR (Fig 7).

### Detection of clinical samples

In all, 103 specimens from farms were assessed by LAMP, CPA, IMSA and real-time PCR for the LT-II gene, respectively. As shown in Table 3, nine of the 103 clinical strains were positive by IMSA, and 94 strains were negative. This was consistent with LAMP, CPA and real-time PCR data. Therefore, IMSA reliably detected clinical samples (S1–S4 Figs).

**Table 3:** Detection results for clinical samples.

Detection methods	Porcine diarrheal specimens (n = 63)		Bovine diarrheal specimens (n = 40)		total
	Positive	Negative	Positive	Negative	
LAMP	5	58	4	36	103
CPA	5	58	4	36	103
IMSA	5	58	4	36	103
qPCR	5	58	4	36	103

## Discussion

Infections by ETEC constitute the second main cause of death in pediatric patients of underdeveloped nations[15]. In industrialized countries, ETEC represents an important etiological agent of traveler’s disease[16]. When ETEC was firstly found to infect humans, multiple studies attempted to explore the associated pathogenetic mechanisms and develop detection methods for ETEC strains and related enterotoxins. Since 1983 when LT-II was first isolated by Green et al. [17], there had been few studies and reports on LT-II toxin gene in the past 30 years. Until recently years, the enterotoxin family had again been highlighted as the reported cause of severe diarrhea in calves, especially in developing countries [14]. At present, PCR as a main identification method is commonly used for LT-II gene detection [18]. However, PCR techniques require specialized equipment and are not suitable for small laboratories. Even though techniques overcoming some methodological and cost challenges of PCR while amplifying target sequences similar to PCR have been developed, they still employ relatively complex protocols that require many enzymes and/or specific reagents. Particularly, such techniques require a high temperature denaturation step and/or enzyme-dependent nicking and strand displacement. Indeed, PCR based methods depend upon expensive equipment and skilled technicians, which limits their application in field deployment. In order to avoid these obstacles, many easy-to-use isothermal amplification detection methods have been developed. Isothermal amplification of DNA under the field environment is feasible, convenient and fast. Compared with PCR-related techniques, they are less susceptible to DNA or RNA contaminants in specimens [19]. It was shown that *Bst* polymerase employed in isothermal amplifications is more tolerant of inhibitory molecules in comparison with Taq polymerase employed for qPCR [20]. Thus, the DNA used in isothermal amplification assays does not need the extent of purification required for real-time PCR.

Since 2000, LAMP has been broadly employed to detect a variety of pathogenic microorganisms. Subsequently, CPA, another isothermal amplification method, was firstly used to detect *Mycobacterium tuberculosis* in sputum specimens [9, 21]. Similar to LAMP, CPA is based on amplification using many cross-linked primers. Recently, IMSA was proposed, with the working principle illustrated. This method has been used for rapidly detecting Human Enterovirus 71 (EV71) and Coxsackievirus A16 (CVA16) by Ding and collaborators [12].

Here, IMSA’s diagnostic performance as compared with those LAMP and CPA. Sensitivity data showed that IMSA had a wider detection range for LT-II with a limit of detection two times lower than that of LAMP, CPA and real-time PCR. In addition, IMSA consumed less time than CPA. Moreover, the specificity of IMSA was very good because of its three primer pairs that can help detect any LT-II^+^ strain while not cross-reacting with related control strains. A total of nine of the 103 clinical specimens tested in the current study were shown to be positive by IMSA, which was similar to CPA, LAMP and real-time PCR results (S1–S4 Figs). These findings demonstrated that the reliability of IMSA makes it a potential tool for clinical application. One of the biggest problems of molecular diagnostics is cross-contamination, mainly caused by aerosols. To avoid such contamination, separate rooms, operation under fume hoods and sample addition with filter pipette tips have been used in the laboratory. However, these operations not only increase costs but also limit the application range. In this study, we used a special separation tube (Hua-Feng tube) to prevent amplicon or aerosol leakage. In these conditions, the reaction mixture could turn green when positive (SYBR Green I) or would remain orange (negative), which makes it easy to assess samples by color change. Such an approach overcomes multiple challenges of other methods, including elevated cost, complexity and low specificity.

In conclusion, we developed and assessed IMSA for rapid detection of LT-II^+^ ETEC, which is a common pathogen responsible for an economically important disease. This method is a rapid and very reliable tool that can be used by poorly equipped laboratories or in the wild. The evaluated IMSA method for LT-II gene detection has the potential to be used even by mobile laboratories and small diagnostic units.

## Supporting information

S1 Fig Results of clinical samples assessed by the LAMP method. A, D, G, J and M, Agarose gel electrophoresis for LAMP reactions; B, E, H, K and N, Direct visualization by the naked eye after staining with SYBR Green I for LAMP reactions; C, F, I, L and O, Visualization under UV light after staining with SYBR Green I for LAMP reactions. M, Trans 2K plus II DNA marker; N, negative control. P, LT-II^+^ positive control; 1–103: Clinical samples.
(TIF)

S2 Fig Results of clinical samples assessed by the CPA method. A, D, G, J and M, Agarose gel electrophoresis for CPA reactions; B, E, H, K and N, Direct visualization with the naked eye after staining with SYBR Green I for CPA reactions; C, F, I, L and O: Visualization under UV light after staining with SYBR Green I for CPA reactions. M, Trans 2K plus II DNA marker; N, negative control. P, LT-II^+^ positive control; 1–103, Clinical samples.
(TIF)

S3 Fig Results of clinical samples assessed by the IMSA method. A, D, G, J and M, Agarose gel electrophoresis analysis for IMSA reactions; B, E, H, K and N, Direct visualization with the naked eye after staining with SYBR Green I for IMSA reactions; C, F, I, L and O, Visualization under UV light after staining with SYBR Green I for IMSA reactions. M, Trans 2K plus II DNA marker; N, negative control. P, LT-II^+^ positive control; 1–103, Clinical samples.
(TIF)

S4 Fig Results of clinical samples assessed by the real-time PCR method. P, LT-II^+^ positive control.
(TIF)
