# Supplementary figures and images for: Establishment and application of isothermal multiple-self-matching-initiated amplification (IMSA) in detecting Type II heat-labile enterotoxin of Escherichia coli
Source: PLoS One. 2019 May 2;14(5):e0216272. doi: 10.1371/journal.pone.0216272 (PMC6497269; doi:10.1371/journal.pone.0216272)

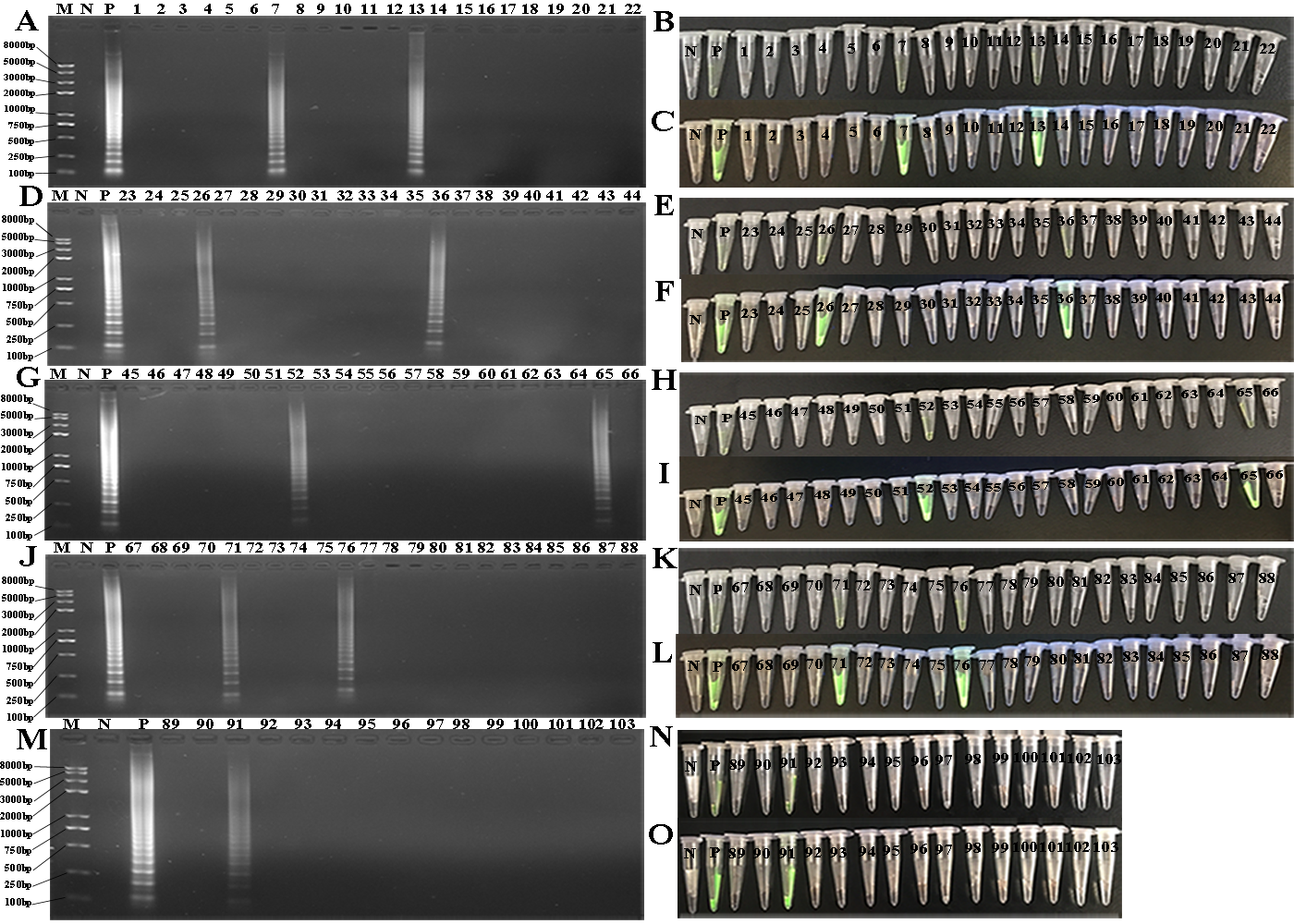

Supplement: S1 Fig — A, D, G, J and M, Agarose gel electrophoresis for LAMP reactions; B, E, H, K and N, Direct visualization by the naked eye after staining with SYBR Green I for LAMP reactions; C, F, I, L and O, Visualization under UV light after staining with SYBR Green I for LAMP reactions. M, Trans 2K plus II DNA marker; N, negative control. P, LT-II+ positive control; 1–103: Clinical samples. (TIF) [file pone.0216272.s001.tif]

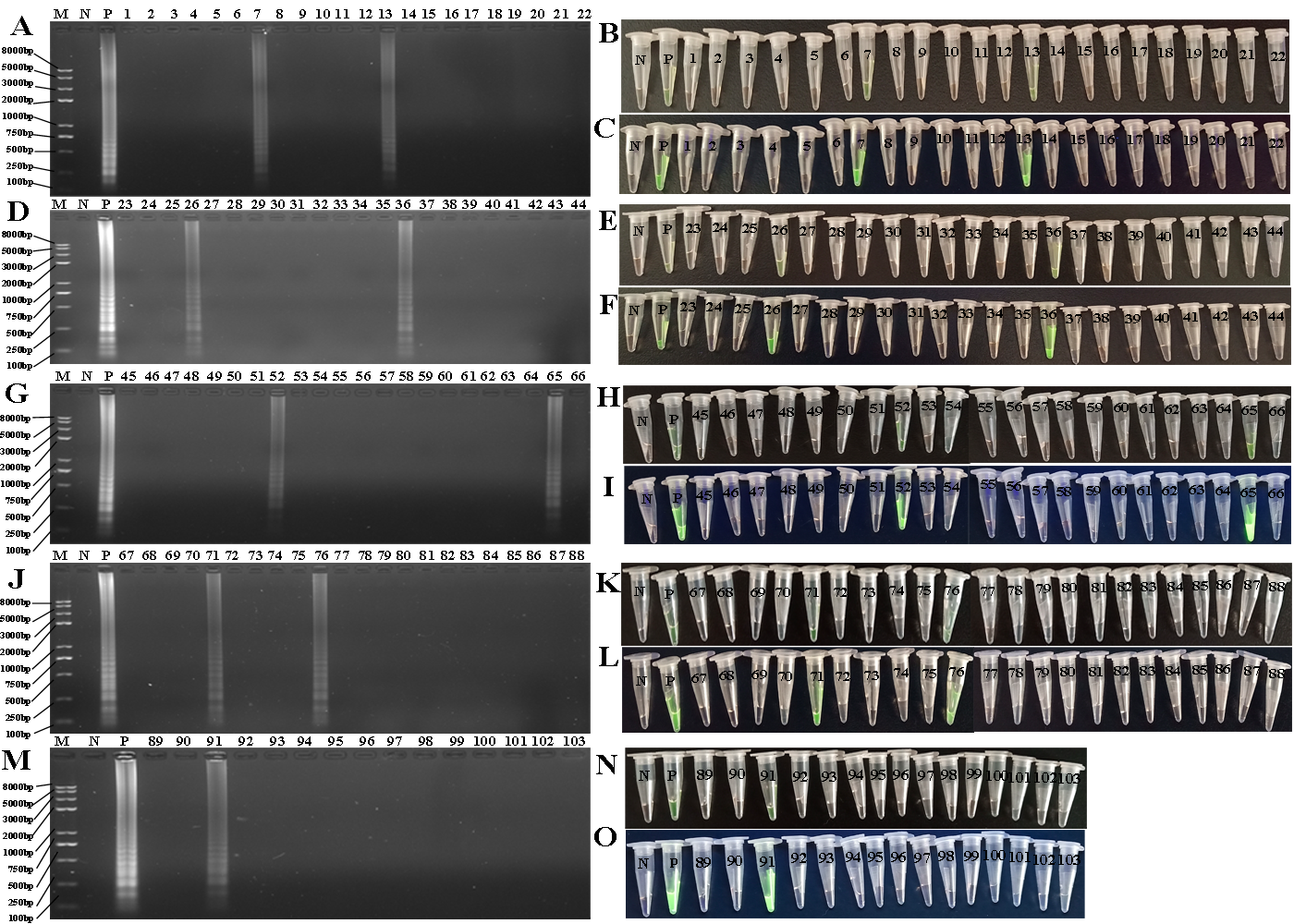

Supplement: S2 Fig — A, D, G, J and M, Agarose gel electrophoresis for CPA reactions; B, E, H, K and N, Direct visualization with the naked eye after staining with SYBR Green I for CPA reactions; C, F, I, L and O: Visualization under UV light after staining with SYBR Green I for CPA reactions. M, Trans 2K plus II DNA marker; N, negative control. P, LT-II+ positive control; 1–103, Clinical samples. (TIF) [file pone.0216272.s002.tif]

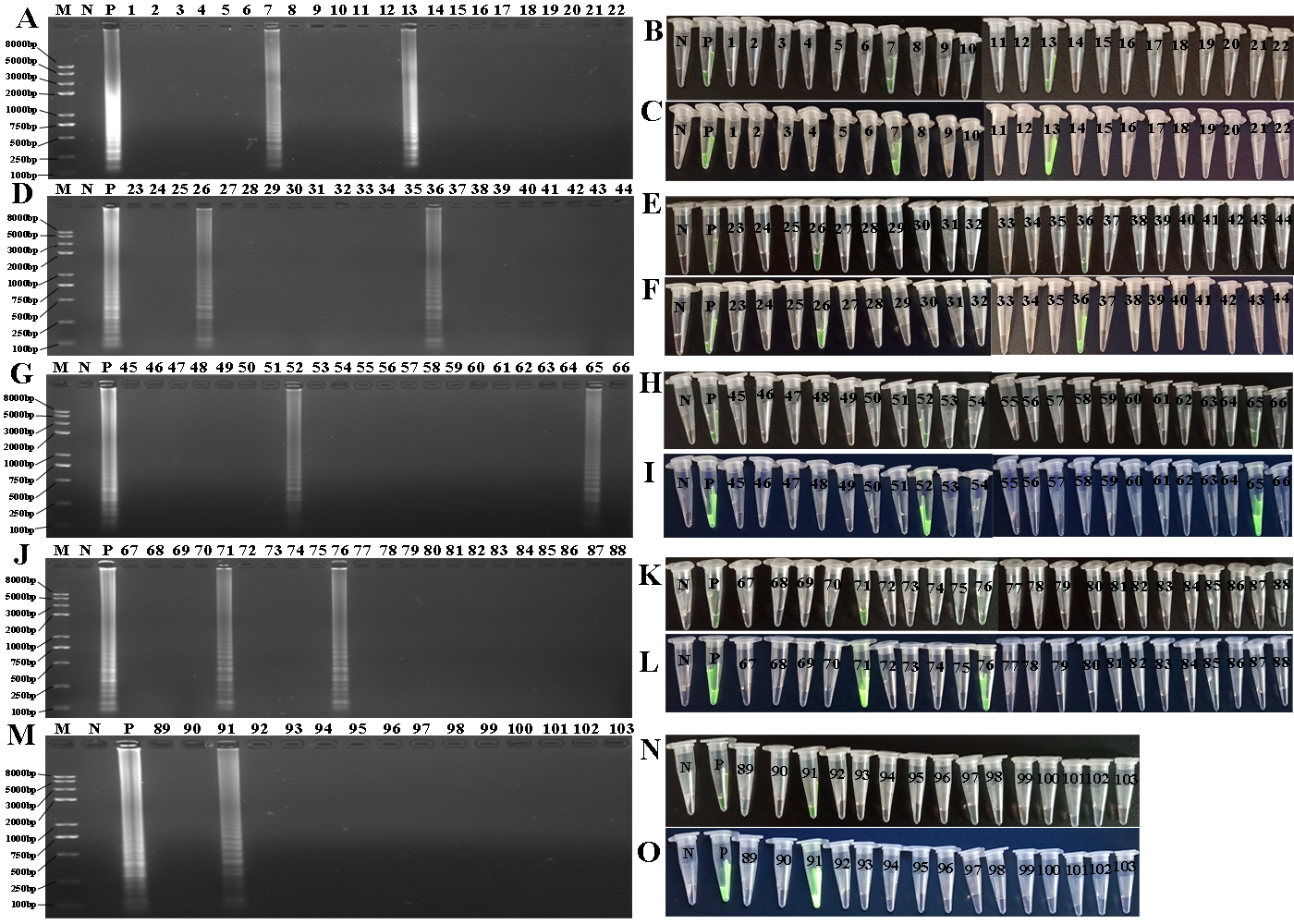

Supplement: S3 Fig — A, D, G, J and M, Agarose gel electrophoresis analysis for IMSA reactions; B, E, H, K and N, Direct visualization with the naked eye after staining with SYBR Green I for IMSA reactions; C, F, I, L and O, Visualization under UV light after staining with SYBR Green I for IMSA reactions. M, Trans 2K plus II DNA marker; N, negative control. P, LT-II+ positive control; 1–103, Clinical samples. (TIF) [file pone.0216272.s003.tif]

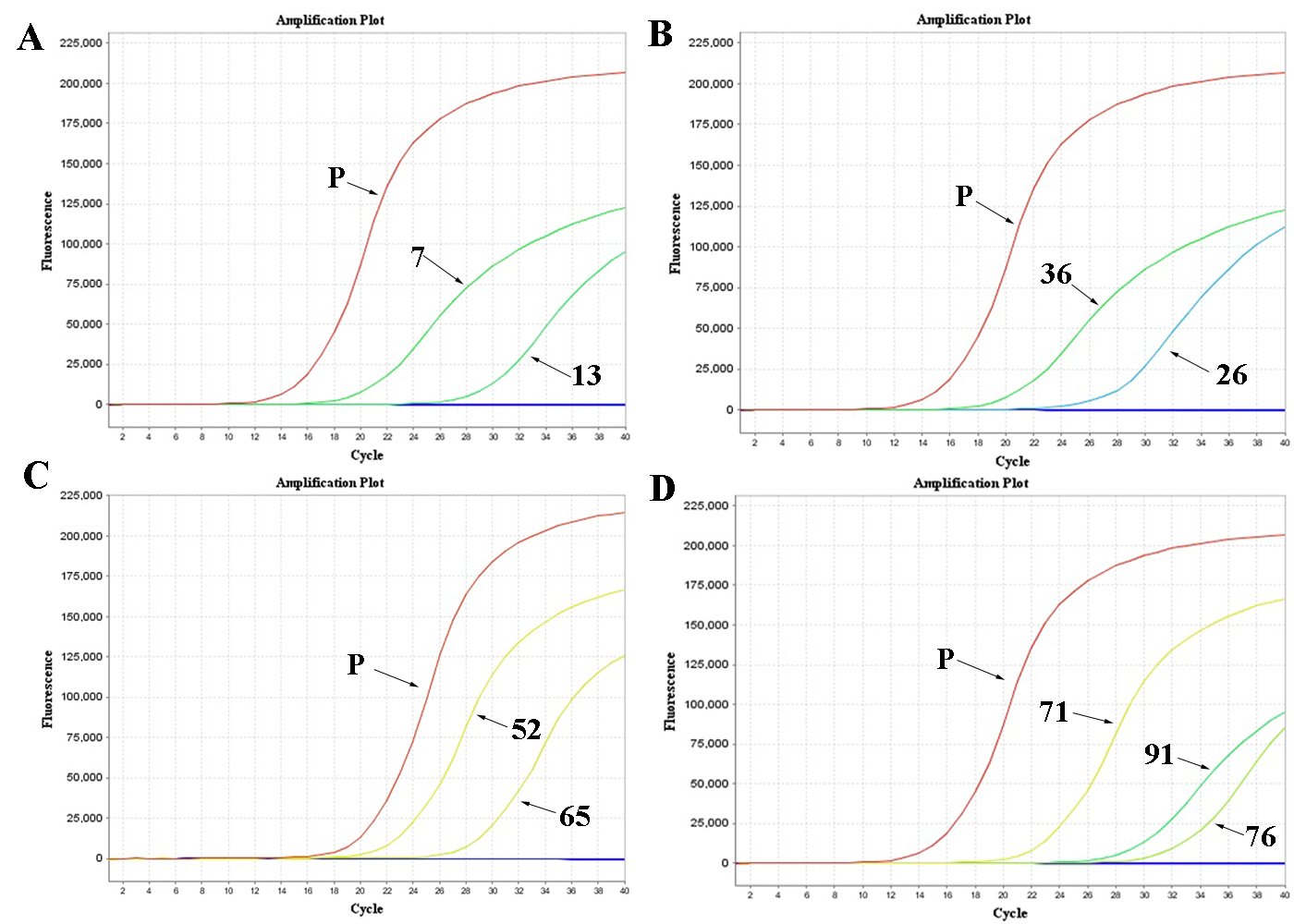

Supplement: S4 Fig — P, LT-II+ positive control. (TIF) [file pone.0216272.s004.tif]
